# A swallowed denture leading to misdiagnosis with esophageal neoplasm: a case report

**DOI:** 10.1093/omcr/omae047

**Published:** 2024-07-09

**Authors:** Roozbeh Shadidi Asil, Amir Zamani, Zahra Fooladi, Kasra Hatampour

**Affiliations:** Department of General Surgery, Loghman Hakim Hospital, Shahid Beheshti University of Medical Sciences, Tehran, Iran; Department of General Surgery, Loghman Hakim Hospital, Shahid Beheshti University of Medical Sciences, Tehran, Iran; Department of Radiology, Rasoul Akram Hospital, Iran University of Medical Sciences, Tehran, Iran; Department of General Surgery, Loghman Hakim Hospital, Shahid Beheshti University of Medical Sciences, Tehran, Iran

**Keywords:** Esophageal foreign bodies, Dysphagia, Endoscopy, Dentures, Misdiagnosis, Case Report

## Abstract

**Introduction:**

Foreign body ingestion can lead to esophageal complications, including perforation and impaction, in up to 20% of cases, making it a critical situation. Misdiagnosis or delayed diagnosis can cause severe complications.

**Case presentation:**

We present the case of a 78-year-old female who swallowed an acrylic partial denture leading to progressive dysphagia and a vegetative ulcerative lesion on endoscopy. The lesion was initially misdiagnosed as a neoplasm of the esophagus. CT scan and a repeat endoscopy revealed the presence of a denture in the esophagus. The denture was successfully removed with a rigid esophagoscope, and no evidence of complications was reported in follow-up visits.

**Discussion:**

Diagnosis of esophageal foreign bodies involves imaging studies and endoscopy, which is the gold standard for diagnosis and management. CT scans also have an important role in diagnosing controversial cases. Treatment depends on the size, shape, and location of the object.

## INTRODUCTION

Foreign body ingestion is a common occurrence, particularly among the elderly, pediatrics, those with psychiatric disorders or developmental delays, and individuals with alcohol intoxication [1]. 10%–20% of esophageal foreign bodies need intervention and the rest pass spontaneously. Up to 50% of these ingestions are misdiagnosed with other situations [2]. The clinical presentation of foreign body ingestion can range from asymptomatic to life-threatening, depending on various factors such as the texture, size, and location of the ingested object. Esophageal foreign bodies are particularly concerning, as they can cause complications such as perforation, obstruction, and stricture formation [3]. While more than 80% of swallowed foreign objects pass without intervention, the esophagus is the most common site of impaction in both adults and children [4, 5]. A normal radiogram cannot rule out this diagnosis, particularly in patients who aren’t able to express their experience properly due to cognitive issues like dementia, particularly in the older ages. Also, radiolucent dentures, which are frequently the case with modern dentures, cannot be seen in the radiogram. For this reason, some cases may be misdiagnosed. While it is not unusual for foreign bodies to be swallowed, it is rare for them to remain lodged for a period of two months [6].

In this case report, we describe the case of a 78-year-old female who accidentally swallowed an acrylic partial denture, resulting in progressive dysphagia and a vegetative ulcerative lesion on endoscopy (initially misdiagnosed as a neoplasm of the esophagus). We hope that this report will contribute to the understanding and management of similar cases in the future.

## CASE REPORT

A 78-year-old female with a past medical history of hypertension and diabetes mellitus presented to the surgical clinic with a two-month history of progressive dysphagia. The dysphagia began with solid foods and progressed to liquids and was accompanied by occasional chest pain and regurgitation. The dysphagia started suddenly. The patient also reported a 6-kilogram weight loss during this period. However, she didn’t complain about cough, fever, or gastrointestinal bleeding. Due to the patient’s old age and decreased awareness, we presumed that the history of her present illness was not reliable. The patient had previously undergone an upper gastrointestinal endoscopy at another medical center two weeks after her symptoms started, including dysphagia, which revealed a vegetative ulcerative lesion in the cervical esophagus, located 20 cm from the incisors, thought to be malignant. Biopsy specimens were taken from the ulcer margin, and the pathology report after two weeks confirmed the presence of esophageal ulceration without any signs of atypia or malignancy.

**Figure 1:**
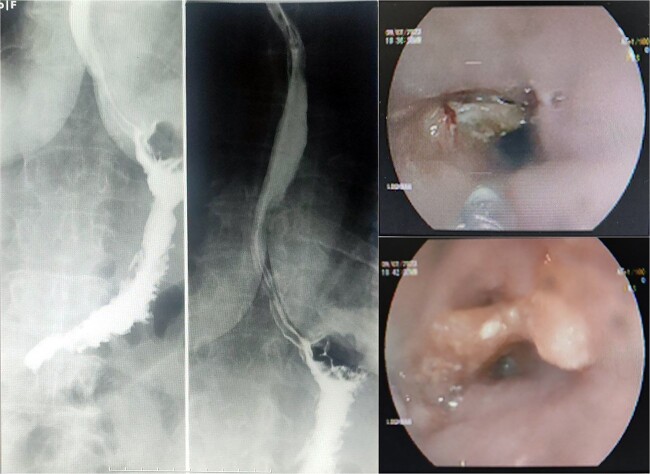
Barium swallow esophagogram shows no signs of malignancy or filling defect (Left image). Also, upper endoscopy revealed a stuck object inside the esophageal wall.

**Figure 2:**
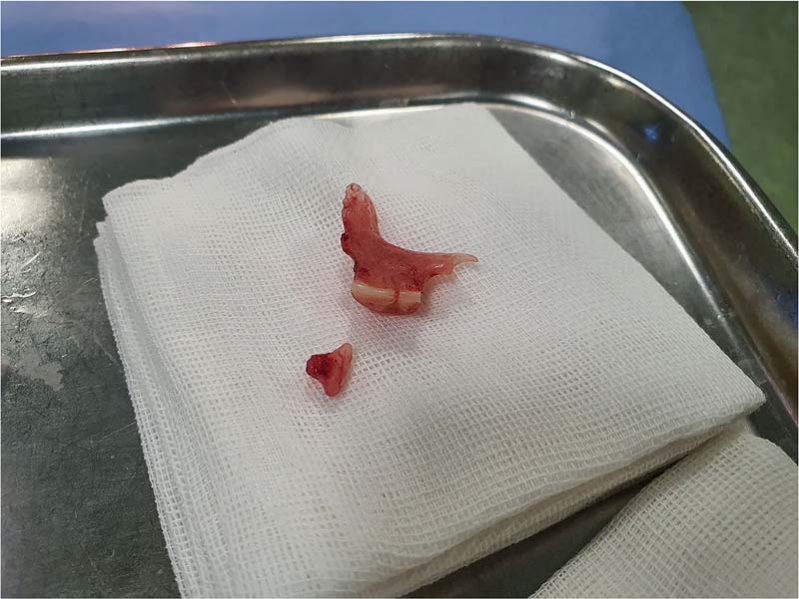
The object was removed successfully. As you see, this is an acrylic partial denture.

Upon being admitted to our center for surgical purposes, we first decided to do more investigations to rule out malignancies, so a chest X-ray was obtained, revealing no pathological findings. A barium swallow study was also performed but did not demonstrate any filling defects in the esophagus. Then a thoracoabdominal CT scan with IV and Oral contrast was performed. The CT scan was not suggestive of an esophageal neoplasm at all. The patient underwent a repeat endoscopy which revealed the presence of a denture in the esophagus (Fig. 1). Attempts to remove the denture using a snare and forceps were unsuccessful due to the size and shape of the object. Consequently, a decision was made to excise the denture with a rigid esophagoscope, which was ultimately successful (Fig. 2). The procedure was completed without any complications, and the patient’s dysphagia and other symptoms resolved after the operation. The patient was discharged home after a 3-day hospital stay with instructions to follow up with her primary care physician.

During follow-up visits, the patient reported no recurrence of symptoms and showed no evidence of esophageal stricture or other complications. The patient continued to follow up regularly with her primary care physician and surgeon.

This work has been reported in line with the SCARE criteria [7].

## DISCUSSION

Foreign body ingestion is a common occurrence, particularly in the elderly population, and can lead to a range of clinical presentations, from asymptomatic to life-threatening which is a medical urgent [8]. Some foreign bodies may be buried in the mucosal or muscular layer of the esophagus causing mucosal edema, tracheal compression, mucosal ulceration, infections like para or retropharyngeal abscess, mediastinitis. The management of esophageal foreign bodies can be challenging and is associated with complications such as perforation, obstruction, and stricture formation [9]. Up to 80% of cases can be managed conservatively, but some may require surgical intervention. In this case, the patient was presented with sudden and progressive dysphagia, weight loss, and a vegetative ulcerative lesion that mimics a neoplastic reason. Esophageal foreign bodies are mostly located at the level of the cricopharyngeal muscle because it is the narrowest portion of the esophagus [10].

Diagnostic evaluation of esophageal foreign bodies usually involves imaging studies such as barium swallow or computed tomography (CT) scan. Endoscopy is the gold standard for diagnosis and management of esophageal foreign bodies, with a success rate of over 95%. In this case, the patient underwent an upper gastrointestinal endoscopy in another center, which revealed a vegetative ulcerative lesion in the cervical esophagus, misdiagnosed as a neoplasm of the esophagus. Then in our center first we perform a thoracoabdominal CT scan with IV and Oral contrast which hand no signs of malignancies. So, it raised our suspicions about the first endoscopy, and we repeated it, and this time the diagnosis was performed.

Treatment of esophageal foreign bodies depends on the size, shape, and location of the object [8]. In this case, attempts to remove the denture with a snare and forceps were unsuccessful. A decision was made to excise the denture with a rigid esophagoscope, which was successful.

Complications of esophageal foreign bodies can include perforation, obstruction, and stricture formation. Prompt diagnosis and appropriate management are crucial to prevent these complications. In this case, the patient’s symptoms resolved after the procedure, and no evidence of esophageal stricture or other complications was reported in follow-up visits.
